# Metaheuristic-based gallstone classification using rotational forest explained with SHAP

**DOI:** 10.3389/fdgth.2025.1727559

**Published:** 2026-01-16

**Authors:** Keshika Shrestha, Proshenjit Sarker, Jun-Jiat Tiang, Abdullah-Al Nahid

**Affiliations:** 1 Electronics and Communication Engineering Discipline, Khulna University, Khulna, Bangladesh; 2 Centre for Wireless Technology, CoE for Intelligent Network, Faculty of Artificial Intelligence and Engineering, Multimedia University, Cyberjaya, Selangor, Malaysia

**Keywords:** bald eagle search, gallstone, machine learning, rotational forest classifier, SHAP

## Abstract

**Introduction:**

Cholelithiasis, commonly known as Gallstone disease, occurs when hardened deposits form in the gallbladder or bile ducts. It affects millions of people worldwide and is especially common in women. While many people may not experience any symptoms, symptomatic cases can present with acute cholecystitis and other complications such as pancreatitis and even gallbladder cancer. However, this disease presents a clinical challenge due to its variable symptoms and risk of serious complications. Therefore, early prediction of gallstones is essential for timely intervention.

**Method:**

Thus, our study presents a novel approach for predicting gallstones. In this study, we have presented a Rotational Forest (RoF) classifier optimized using the Bald Eagle Search (BES) algorithm for gallstone prediction based on a tabular dataset. Our research has been conducted across two frameworks: using RoF alone and using RoF with the BES algorithm.

**Result:**

While using RoF alone, an accuracy of 78% and an AUC of 0.867 was obtained using all features. An accuracy of 75.78% and an AUC of 0.860 were obtained for RoF with the BES algorithm using only 17 features. SHapley Additive exPlanations (SHAP) and Local Interpretable Model-agnostic Explanations (LIME) analysis has distinguished CRP, Vitamin D, Obesity, HGB, and BM as the most dominant features.

**Discussion:**

Likewise, we have also compared our work with other novel works and validated the performance of our model for the prediction of gallstones.

## 1 Introduction

The gallbladder is a small, pear-shaped organ located beneath the liver that plays a crucial role in the digestive process. Although small in size, it can cause a serious medical condition. The most common one is gallstone disease (cholelithiasis). Gallstones are a common gastrointestinal disease affecting millions worldwide. They are hardened deposits of cholesterol or bilirubin that can develop without presenting any symptoms (1). Globally, gallstone disease affects approximately 10%–20% of adults, hence making it a significant public health concern (2). In the research done by Barbara et al. (3), it was found that among 132 patients ranging from 18 to 65 years of age, 6.7% of men and 14.6% of women were affected by gallstones. Stinton and Shaffer (4) reported that the occurrence of gallstones is influenced by a range of factors such as age, sex, diet, and underlying medical conditions such as obesity and diabetes, while Novacek (5) documented that sex hormones were also responsible for this disease. Some other risk factors, as reported by Attili et al. (6) include body mass index as well as maternal family history of gallstone disease. According to the research done by Heaton et al. (7), 80% of gallstones are asymptomatic, and although asymptomatic can lead to serious complications such as bile duct obstruction, pancreatitis. The main preferred treatment of gallstone disease is laparoscopic cholecystectomy (6). However, it is not without risk or cost, as many individuals with gallstones remain asymptomatic throughout their lives. Performing surgery in such cases exposes patients to overtreatment, increased healthcare expenditure, and overall discomfort. All of these can significantly increase morbidity. Thus, early and accurate prediction of gallstones can play a vital role in preventing severe outcomes as well as easing the burden on patients.

The accurate prediction of gallstones remains a challenge due to the complex interrelation between factors like genetics, metabolism, diet, lifestyle, hormones, etc. This is where machine learning (ML) presents an effective approach for accurate prediction. In recent years, ML has proved to be a powerful tool in the field of medical diagnostics as it uncovers hidden patterns within clinical datasets. Studies such as those by Rajkomar et al. (8) and Choi et al. (9) have demonstrated the potential of ML algorithms to predict clinical outcomes. Several studies have attempted to apply traditional statistical or ML methods, such as logistic regression, decision trees, and random forests, for the prediction of gallstones. The research done by Deng et al. (10) employed four algorithms—Logistic Regression, Gaussian Naive Bayes (GNB), Multi-Layer Perceptron (MLP), and Support Vector Machine (SVM) to predict the risk factors for gallstone formation, where logistic regression achieved an accuracy of 69.9%. Its findings were limited by sample size, population specificity, and lacked external validation. Similarly, Esen et al. (11) reported an accuracy of 85.42% for the early prediction of gallstone disease. However, their model used a high number of features (38 features), which will be both time-consuming and resource-intensive. A study by Ma et al. (12) employed decision tree, logistic regression, etc., for the prediction and identification of patients at high risk for acute gangrenous cholecystitis. Another study utilized logistic regression and decision trees to analyze dietary patterns and their association with gallstone risk (13). All these studies were often constrained by class imbalance, lack of advanced optimization, external validation, as well as the limited focus on interpretability. To address these limitations of prior studies, we have introduced an optimized Rotational Forest (RoF) with SHapley Additive exPlanations (SHAP) analysis for the prediction of gallstones.

Among various ensemble methods, RoF was selected for our study as it has shown potential to generate diverse and accurate classifiers. RoF enhances diversity by applying feature extraction through principal component analysis, unlike Random Forest or XGBoost, which primarily rely on random feature sampling (14). RoF classifier has shown superior performance in various medical contexts, including the study done by Aličković and Subasi (15) on breast cancer prediction, which obtained an accuracy of 99.48%. Likewise, another research on liver disease classification using RoF attained an accuracy of 72.7% (16). Similarly, this ensemble method was also used for cancer classification due to its superior performance (17). The RoF outperformed other ensemble methods in accuracy (76%) and F-measure (75%) when it was used for the prediction of Parkinson's disease severity (18). Bharti et al. (19) obtained an accuracy of 96.6% using an ensemble with RoF for the analysis of liver fibrosis staging. This clearly validates the improved performance of the classifier in medical datasets. Despite its strengths, RoF still depends heavily on optimal parameter selection and feature subset quality. As mentioned in the study done by Budnik and Krawczyk (20), the performance of RoF varies significantly with feature-subspace settings. It can limit the predictive power of RoF if these challenges are poorly addressed.

To overcome the constraints of RoF, our study incorporates a metaheuristic algorithm to optimize the RoF model. Metaheuristic algorithms are highly optimized methods that employ intelligent search strategies to find near-optimal solutions in complex problems. The Bald Eagle Search (BES) algorithm is one of the nature-inspired metaheuristic algorithms based on the intelligent hunting behavior of bald eagles. The BES algorithm fine-tunes parameters and improves feature selection. This property of the algorithm enhances any model's overall performance and robustness (21). The BES demonstrates a strong balance between exploration and exploitation and also enables faster convergence and more reliable optimization outcomes in comparison to other metaheuristics such as Particle Swarm Optimization, Genetic Algorithm, or Whale Optimization Algorithm (22). Due to these factors BES algorithm was chosen for the optimization of the RoF classifier.

The most important element in the context of applying ML models to medical datasets is that it should be easy to understand, so that it can be trusted and used responsibly in healthcare. Therefore, to address this need and bridge the gap between prediction and clinical interpretability, our study employs SHAP. SHAP analyzes and explains feature contributions of the dataset for the prediction of gallstones. This ensures that the model not only predicts accurately but also offers clear and understandable insights into the rationale behind each decision. SHAP also aims to explain why the model made a particular decision by linking features to the outcomes, unlike other explainability frameworks such as LIME. This makes it especially suitable for clinical decision support.

Hence, this research aims to analyze the performance of the BES algorithm with the RoF classifier using a tabular dataset with 319 instances with 38 numerical features. Our approach holds a broader impact for healthcare in addition to its technical precision. By enabling early prediction of gallstones, our model can reduce unnecessary surgical interventions. This will, in turn, lower healthcare costs and improve patients’ quality of life. Furthermore, our framework can also be extended to other gastrointestinal and metabolic disorders, making it scalable and versatile. In this study, we present a novel, interpretable, and optimized RoF framework for predicting gallstones. Our aim is to support healthcare decisions and reduce unnecessary surgical interventions.

## 2 Materials and methods

**Figure 1:**
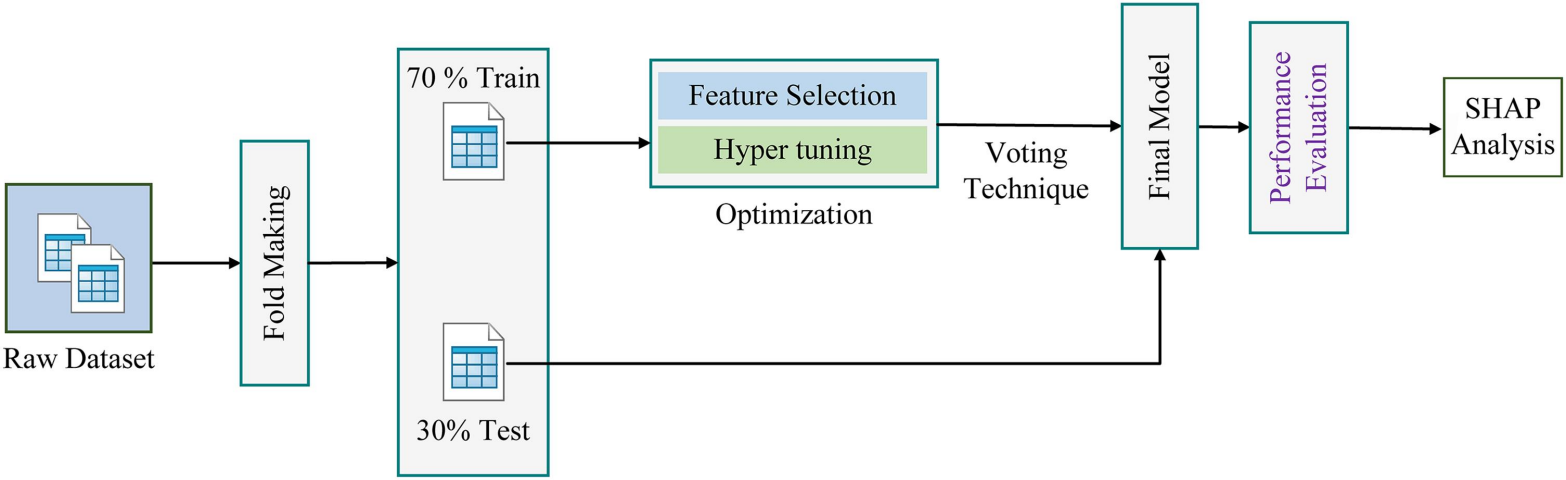
Overview of the methodology.

For our work, we have collected the raw gallstone dataset from the UCI machine learning repository and prepared it by creating appropriate data folds. The dataset was then partitioned into 70% for training and 30% for testing. The RoF algorithm was employed to train the model, and the training set was further optimized using the BES algorithm. Then the final model was created by combining both the optimized model and the test dataset. Finally, SHAP was introduced to enhance the interpretability of the model. An overview of the proposed methodology is presented in Figure 1.

### 2.1 Raw dataset

The dataset utilized in this study was obtained from the UCI machine learning repository. This dataset can be found at URL: https://archive.ics.uci.edu/dataset/1150/gallstone-1. This dataset is particularly designed for the prediction of gallstone occurrence. It uses medical and demographic data from 319 individuals, and each instance in the dataset represents a unique patient record described by 38 numerical features. The dataset is entirely numeric and eliminates the need for encoding into categorical variables. This dataset was obtained over the course of 1 year, from June 2022 to June 2023. Among the 319 individuals, 162 were male and 157 were female, with females having a higher number of gallstone cases. The participants ranged in age from 18 to 90 years. According to the World Health Organization's BMI classification, about 40% of individuals were overweight, 30.5% had a normal weight, 26.5% were classified as obese, and 3% were underweight. Regarding existing health conditions, 43 individuals had been diagnosed with diabetes, while 276 did not report having the condition.

### 2.2 Fold making

**Figure 2:**
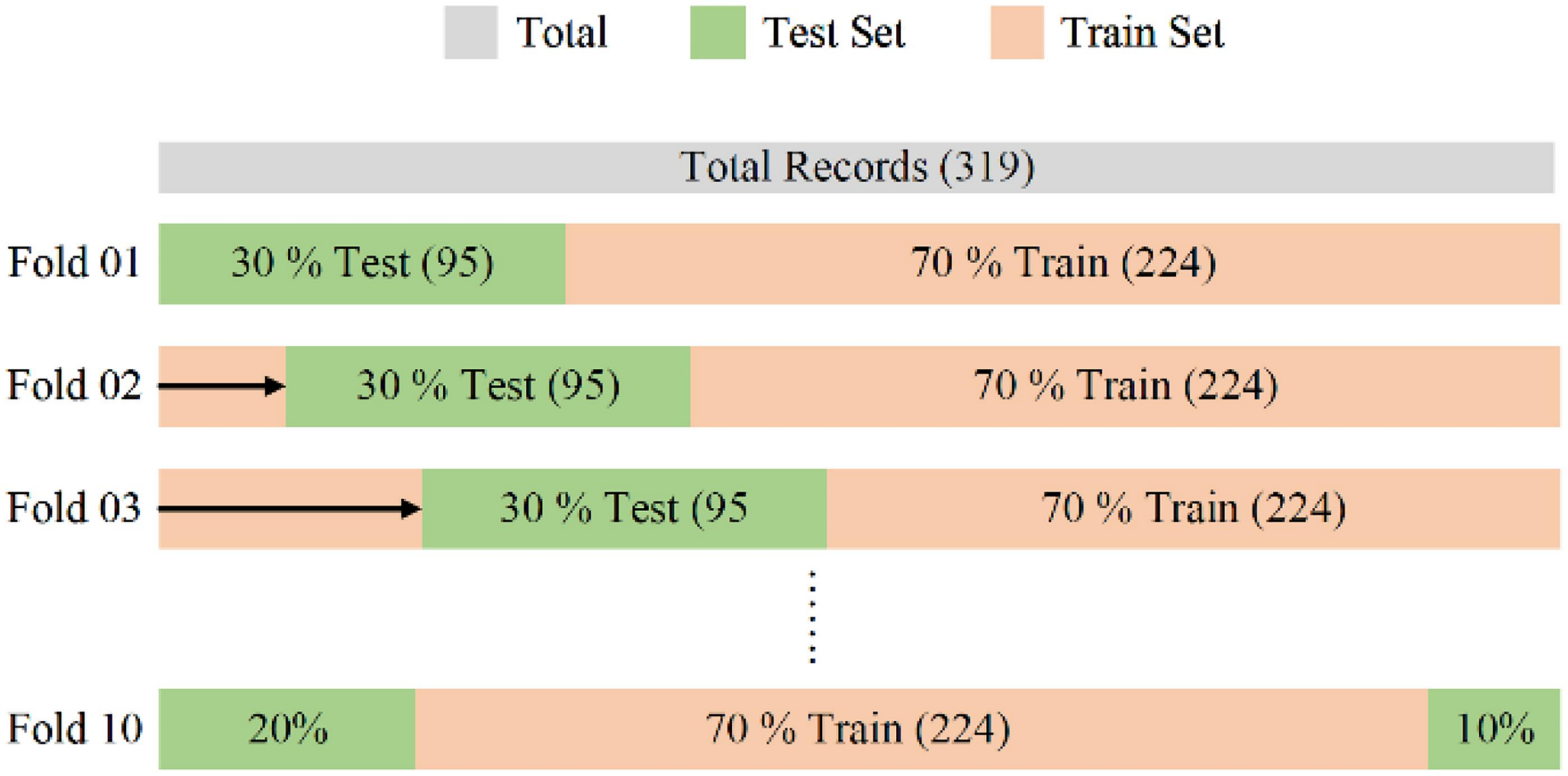
Fold making of the gallstone dataset.

The dataset was divided into subsets or folds for cross-validation. In this process, the dataset has been divided into 10 equally sized folds with proper shuffling to ensure randomness. Each fold consists of two partitions of the total data: 70% for training (224 samples) and 30% for testing (95 samples) as shown in Figure 2. Instead of relying on the default k-fold settings, we have employed a custom fold-making strategy to maintain better control over the dataset and evaluation of predictions. A key advantage of this approach is that each fold has been optimized independently. During optimization, only the training portion of each fold has been used, while the corresponding testing portion has remained completely unseen by the model until the final evaluation stage.

### 2.3 Partition of dataset

Following the cross-validation, the dataset was partitioned into 70% for training and 30% for testing. This was done to analyze the predictive capability of our model. Our model was trained using the RoF ensemble method. This method is designed to improve the accuracy and diversity of decision trees in a classification task. This method transforms the feature space before training each tree. Instead of giving every tree the same original data, it first divides the features into random groups. Then, it applies Principal Component Analysis to each group to create a rotated version of the data. These transformed features are combined to form a new dataset, which is used to train each tree in the ensemble. This “rotation” ensures that each tree learns from a different perspective of the same data, encouraging diversity while preserving meaningful information. When making predictions, the individual trees vote, and the final decision is made based on majority rule. It was first introduced by Rodriguez et al. (23) and is well-suited for high-dimensional datasets, such as ours.

### 2.4 Bald eagle search (BES) algorithm

After performing fold-making, optimization was done using the BES algorithm to further refine the model and enhance its performance. This step focuses on improving feature selection and hyperparameter tuning. The BES algorithm is a nature-inspired metaheuristic algorithm that mimics the hunting strategy of bald eagles (21). Inspired by the intelligent social behavior of bald eagles during their hunting process, the algorithm is structured into three key phases: selecting a search area, exploring within that area, and executing a swooping action to capture prey.

#### 2.4.1 Selecting a search area

The bald eagle identifies promising regions within the selected search space where they can hunt their prey. This behavior is mathematically represented by (Equation 1) (21),

$$P_{new},i=P_{best}+a*r(P_{mean}-P_{i})$$ (1)

where,

*α* = controls position changes and ranges between 1.5 and 2.

r = random number with values 0 and 1

$P_{best}$ = current search space based on the position found in previous searche s

$P_{mean}$ = mean position of all previously searched points

$P_{i}$ = current position of the ith eagle.

#### 2.4.2 Exploring the search area

In this phase, the bald eagle searches intensively within the selected region. It moves in moves in a spiral or curved trajectory. This can be mathematically expressed by (Equation 2) (21),

$$P_{i,new}=P_{i}+y(i)*(P_{i}-P_{i+1})+x(i)*(P_{i}-P_{mean})$$ (2)

where,

$P_{i}$ = current position of the eagle

$P_{i+1}$ = position of the next eagle in the population

$P_{i,new}$ = updated position

x(i),y(i) = random numbers uniformly distributed between—1 and 1

#### 2.4.3 Swooping

In the swooping phase, the bald eagle rapidly dives toward the prey. This represents a focused movement toward the best solution found so far. This is mathematically represented in (Equation 3) (21)

$$P_{i,new}=rand\;*\;P_{best\;}+\;x_{1}(i)*(P_{i}-c_{1}*\;P_{mean})+y_{1}(i)*(P_{i}-c_{2}*\;P_{best})$$ (3)

where,

$x_{1}(i),y_{1}(i)$ = additional random numbers that control the impact of swooping behavior

$c_{1},c_{2}$= constants that balance the influence of $P_{mean}\;$and $P_{best\;}$

The BES algorithm proves to be a suitable choice for optimizing the model trained on the gallstone dataset. This algorithm navigates the complex parameter space and fine-tunes the model. It also helps improve classification performance without adding much computational burden (22). Hence, making it both effective and practical.

### 2.5 Final model

The final model is obtained based on the combination of the optimized trained model and the unoptimized test model. For the optimized trained model, a voting-based strategy was applied to select both features and hyperparameters. During each fold of cross-validation, different subsets of features and hyperparameter configurations were obtained. To ensure consistency and robustness, majority voting was employed for feature selection; any feature that appeared in more than 50% of the folds was retained for the final model. Similarly, for hyperparameters, we are using the mean value of all the hyperparameters.

### 2.6 Performance evaluation

For the evaluation of our model, accuracy, F-score, Precision, and Recall have been used. A confusion matrix has also been used for the visualization of True Positive (TP), True Negative (TN), False Positive (FP), and False Negative (FN) of the model, as well as AUC and ROC.

### 2.7 SHAP

SHAP, which stands for SHapley Additive exPlanations, is a powerful and popular method for explaining the predictions of any machine learning model. It is a game theory-based approach that helps understand how each feature in the dataset contributes to a single prediction (24). It's built upon the concept of Shapley values. These values offer both local interpretability and global interpretability. In this study, SHAP is used as it makes our model's decisions easier to understand and more trustworthy. It explains how different features, such as age, diet, or genetics, influence each prediction. This kind of transparency is especially important in healthcare, where decisions impact real lives. SHAP helps build confidence in the model's outcomes by showing which factors mattered most in predicting gallstones. Hence, making it more useful for both clinicians and researchers (25).

## 3 Results

In this section, the outcome of our research has been presented sequentially. Our result section has been separated into three subparts: Optimizer Outcome, Classifier Outcome, and SHAP Analysis.

### 3.1 Optimizer outcome

We implemented a 10-fold cross-validation approach, where each fold was split into 70% for training and 30% for testing. The RoF classifier was integrated with the BES algorithm for optimization, which was performed exclusively on the training datasets. Each training set was sequentially optimized, resulting in the selection of 10 feature sets and the determination of 10 corresponding hyperparameter sets.

**Figure 3:**
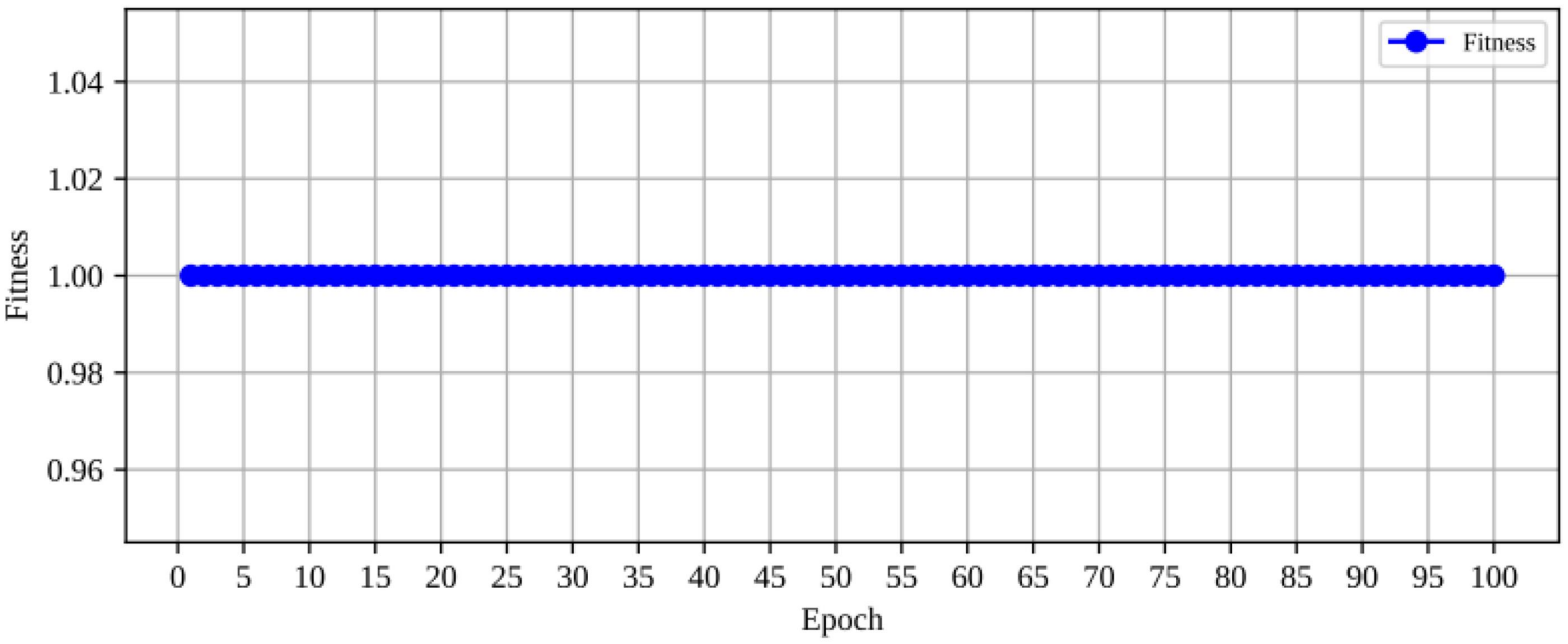
Fitness vs. epoch curve.

When analyzing the fitness vs. epoch curve (Figure 3) during optimization, we observed that the highest fitness value, corresponding to an F1-score of 1, was achieved in the very first epoch. This indicates that the model rapidly converged to an optimal solution.

Table 1 provides a fold-wise breakdown of the selected feature indices and the optimized values of four hyperparameters: n_estimator, min_group, max_group, and remove_proportion. The variations in feature selection and hyperparameter values across folds highlight the model's sensitivity to different data partitions. The final row of the table summarizes the result with a feature set of 17 distinct features [4, 8, 9, 15, 16, 17, 18, 20, 22, 26, 28, 31, 33, 34, 35, 36, 37] and a consistent set if hyperparameters; n_estimator = 118, min_group = 6, max_group = 4, remove_proportion = 0.483. This approach makes sure the final model uses important information from all folds.

**Table 1:** Fold-wise and finalized optimized hyperparameters and selected features.

Optimizer	Fold	Best features	n_estimator	min_group	max_group	remove_proportion
BES	1	(4, 8, 9, 15, 16, 17, 18, 20, 22, 26, 28, 29, 31, 33, 34, 35, 36, 37)	133	4	7	0.486892852
	2	(4, 8, 9, 15, 16, 17, 18, 20, 22, 26, 28, 29, 31, 33, 34, 35, 36, 37)	136	4	7	0.494673715
	3	(4, 8, 9, 15, 16, 17, 18, 20, 22, 26, 28, 31, 33, 34, 35, 36, 37)	102	3	5	0.484244168
	4	(4, 8, 9, 15, 16, 17, 18, 20, 22, 26, 28, 31, 33, 34, 35, 36, 37)	102	3	5	0.484244168
	5	(4, 8, 9, 15, 16, 17, 18, 19, 20, 22, 26, 28, 29, 31, 33, 34, 35, 36, 37)	136	4	7	0.494673715
	6	(4, 8, 9, 15, 16, 17, 18, 20, 22, 26, 28, 29, 31, 33, 34, 35, 36, 37)	133	4	7	0.486892852
	7	(4, 8, 9, 15, 16, 17, 18, 20, 22, 26, 28, 31, 33, 34, 35, 36, 37)	100	3	5	0.485670606
	8	(0, 1, 4, 5, 7, 8, 9, 15, 16, 17, 18, 19, 20, 22, 24, 25, 26, 27, 28, 29, 30, 31, 32, 33, 34, 35, 36, 37)	137	4	7	0.494893893
	9	(4, 8, 9, 15, 16, 17, 18, 20, 22, 26, 28, 31, 33, 34, 35, 36, 37)	100	2	5	0.419243714
	10	(4, 8, 9, 15, 16, 17, 18, 20, 22, 26, 28, 31, 33, 34, 35, 36, 37)	101	4	5	0.500765242
Finalized		(33, 34, 35, 4, 36, 37, 8, 9, 15, 16, 17, 18, 20, 22, 26, 28, 31	118	6	4	0.483219492374505

### 3.2 Classifier outcome

For the classification section, we have organized our research findings into two parts, namely classification performance without optimization and classification performance with optimization.

#### 3.2.1 Classification performance without optimization

The RoF classifier was first evaluated without using any optimizers across a 10-fold cross-validation. We assessed the model's performance through various performance metrics such as accuracy, F1 score, recall, precision, confusion matrices, and ROC curve, as well as their computational efficiency. Table 2 summarizes these performance metrics.

**Table 2:** Performance using only RoF classifier with 10-fold cross-validation.

Fold	Train					Test				
	Acc (%)	F1 (%)	Pre. (%)	Rec. (%)	Time (s)	Acc (%)	F1 (%)	Pre. (%)	Rec. (%)	Time (s)
1	100	100	100	100	13.105	85.263	84.782	84.782	84.782	0.008364200592
2	100	100	100	100	5.210	81.052	79.069	75.555	82.926	0.01100683212
3	100	100	100	100	4.267	78.947	78.260	78.260	78.260	0.008346319199
4	100	100	100	100	5.432	77.894	77.419	78.260	76.595	0.00821685791
5	100	100	100	100	4.309	76.842	76.595	83.720	70.588	0.00795340538
6	100	100	100	100	4.748	75.789	71.604	80.555	64.444	0.01169872284
7	100	100	100	100	4.945	71.578	69.662	73.809	65.957	0.009718418121
8	100	100	100	100	4.221	74.736	75	78.260	72	0.01115393639
9	100	100	100	100	5.668	74.736	77.777	75	80.769	0.008701324463
10	100	100	100	100	4.372	83.157	83.333	85.106	81.632	0.009350776672
Average	100	100	100	100	5.6282	78	77.350	79.331	75.795	0.00945

Acc, accuracy; F1, F-score; Pre., precision; Rec., recall; s, seconds.

The classifier consistently achieved perfect scores (100%) across all training folds. This indicates that it was able to fully fit the training data. However, the test performance showed a variety of results across folds. On average, the RoF classifier obtained an accuracy of 78%, F1-score of 77.350%, precision of 79.331% and 75.795% recall on the test sets. In terms of efficiency, the model maintained a mean training time of 5.63 s and a mean testing time of 0.00945 s.

**Figure 4:**
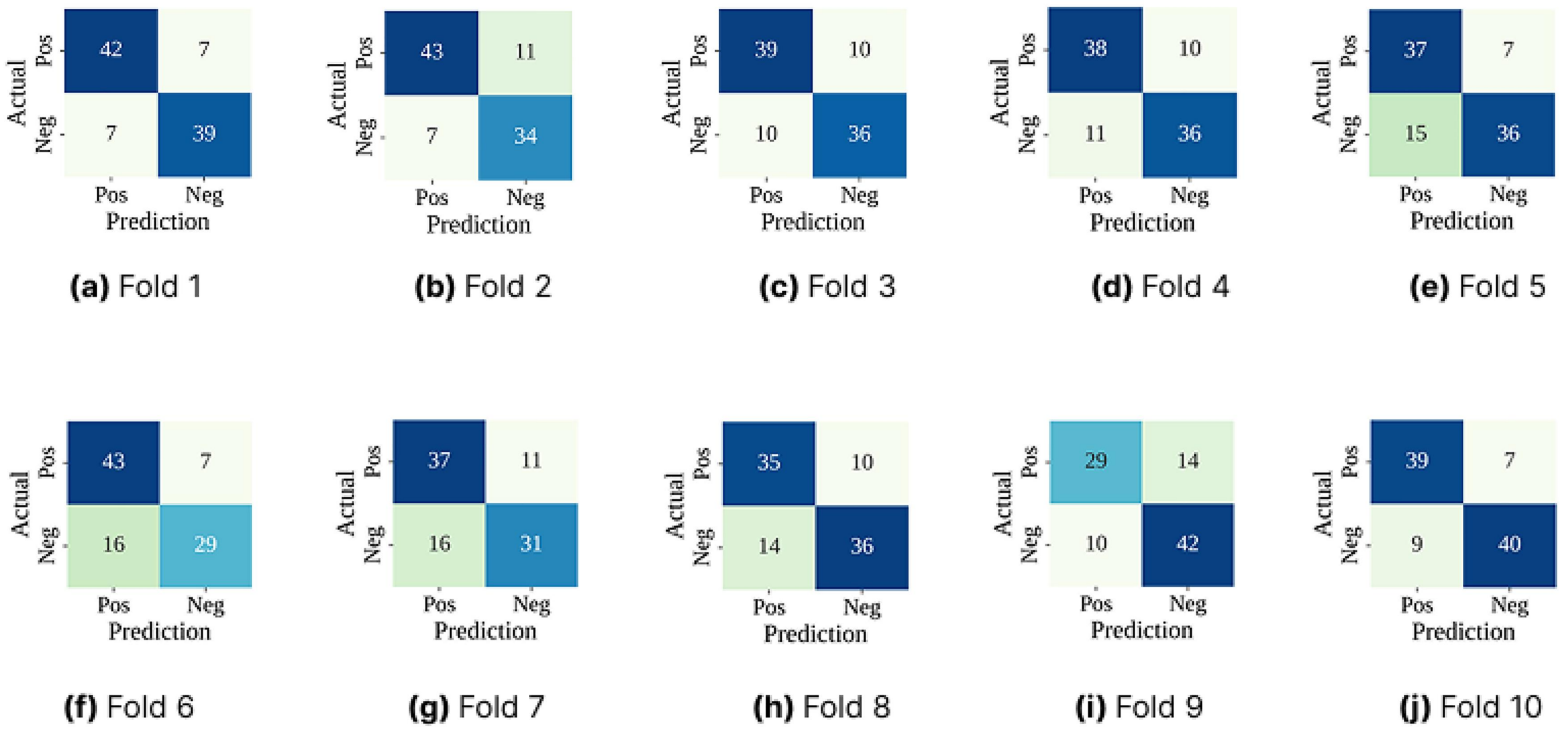
Confusion matrices (CM) across 10-folds using only the classifier with cross-validation, where **(a)** Fold 1 CM, **(b)** Fold 2 CM, **(c)** Fold 3 CM, **(d)** Fold 4 CM, **(e)** Fold 5 CM, **(f)** Fold 6 CM, **(g)** Fold 7 CM, **(h)** Fold 8 CM, **(i)** Fold 9 CM, and **(j)** Fold 10 CM.

The confusion matrices of Figure 4 show that the RoF performed consistently well across all folds. It was able to correctly identify most gallstone and non-gallstone cases, with only a few misclassifications. However, some patients with gallstones were still predicted as healthy (false negatives). In a medical setting, this is important because missing a diagnosis can have serious consequences. This highlights the need for further optimization to make the model more reliable in detecting gallstone cases.

**Figure 5:**
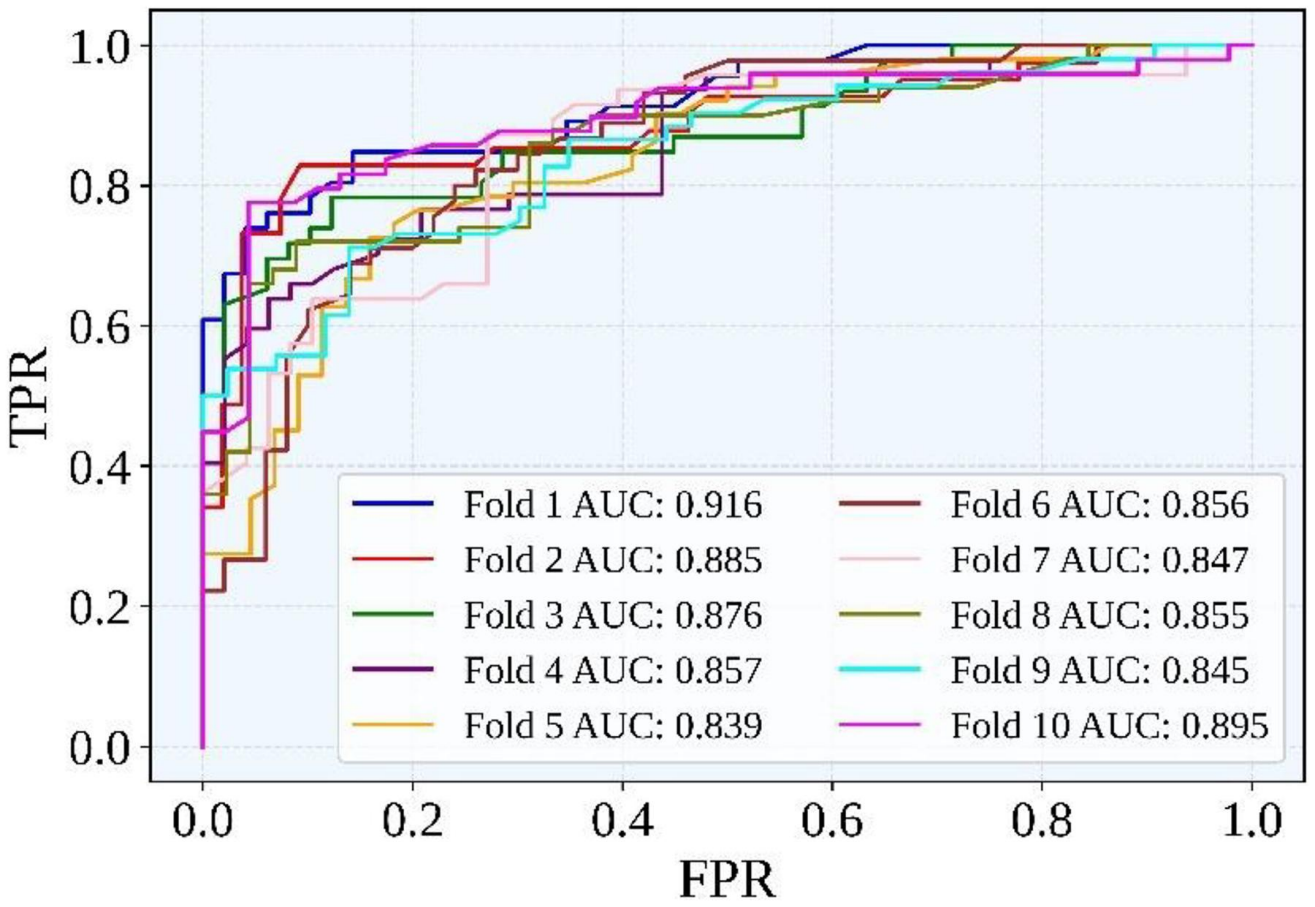
Combined ROC curve of all 10 folds.

Similarly, Figure 5 illustrates the ROC curves of each of the 10 folds. From the figure, it is evident that the baseline RoF was quite good at telling apart patients with gallstones from those without. The model was doing much better than random classification, as the curves stayed well above the diagonal line. The mean AUC of 0.867 further supports the consistent and reliable performance across folds. While these results are promising, they also suggest that additional optimization could further enhance the classifier's ability to distinguish between different categories.

Overall, the baseline RoF classifier showed consistent performance across 10 folds with an average AUC of 0.867. The baseline model also demonstrated stable classification accuracy and efficient training/testing times. However, optimization is necessary to further reduce false negatives and enhance stability across folds. In medical applications such as gallstone prediction, even small improvements in accuracy and reliability are critical. Therefore, the Bald Eagle Search-based optimization was applied to further refine the classifier and achieve more clinically meaningful results.

#### 3.2.2 Classification performance with optimization

The RoF classifier was further evaluated after incorporating BES optimization across 10 folds. The purpose of applying BES optimization was to fine-tune the feature selection and model parameters so that the classifier could achieve better accuracy, more stable results across folds, and efficient computation compared to our baseline model.

During the optimization process, we observed that the selected hyperparameters and features were the same across all folds. The optimization successfully tuned the key hyperparameters, identifying 118 base estimators, a maximum group size of 6, a minimum group size of 4, and a feature removal proportion of 0.483. This process resulted in the selection of 17 highly relevant features for gallstone prediction (Table 1).

Table 3 presents the performance of the RoF-BESA model evaluated through 10-fold cross-validation. The model has consistently achieved perfect training performance across all folds, with 100% accuracy, F1-score, precision, and recall. On the test sets, the model has shown stable and competitive performance, with an average accuracy of 75.789%, F1-score of 75.411%, precision of 76.323%, and recall of 74.700%. The average fold-wise training and testing times were 1.38 s and 0.009464740753 s, respectively.

**Table 3:** Performance using optimized RoF classifier with 10-fold cross-validation.

Fold	Train					Test				
	Acc (%)	F1 (%)	Pre. (%)	Rec. (%)	Time (s)	Acc (%)	F1 (%)	Pre (%	Rec. (%)	Time (s)
1	100	100	100	100	1.59	77.894	76.923	76.086	77.777	0.008745908737
2	100	100	100	100	1.24	76.842	74.418	78.048	71.111	0.007972717285
3	100	100	100	100	1.23	72.631	72.340	73.913	70.833	0.008367776871
4	100	100	100	100	1.76	76.842	77.551	80.851	74.509	0.01091098785
5	100	100	100	100	1.29	69.473	70.707	68.627	72.916	0.01380801201
6	100	100	100	100	1.25	74.736	71.428	66.666	76.923	0.007935762405
7	100	100	100	100	1.22	75.789	74.725	72.340	77.272	0.009310007095
8	100	100	100	100	1.24	77.894	78.350	76	80.851	0.008265256882
9	100	100	100	100	1.21	75.789	77.669	76.923	78.431	0.008763313293
10	100	100	100	100	1.77	80	80	77.551	82.608	0.0105676651
Avg	100	100	100	100	1.38	75.789	75.411	76.323	74.700	0.009464740753

Acc., accuracy; F1, F-score; Pre., precision; Rec., recall; s, seconds; Avg, average.

**Figure 6:**
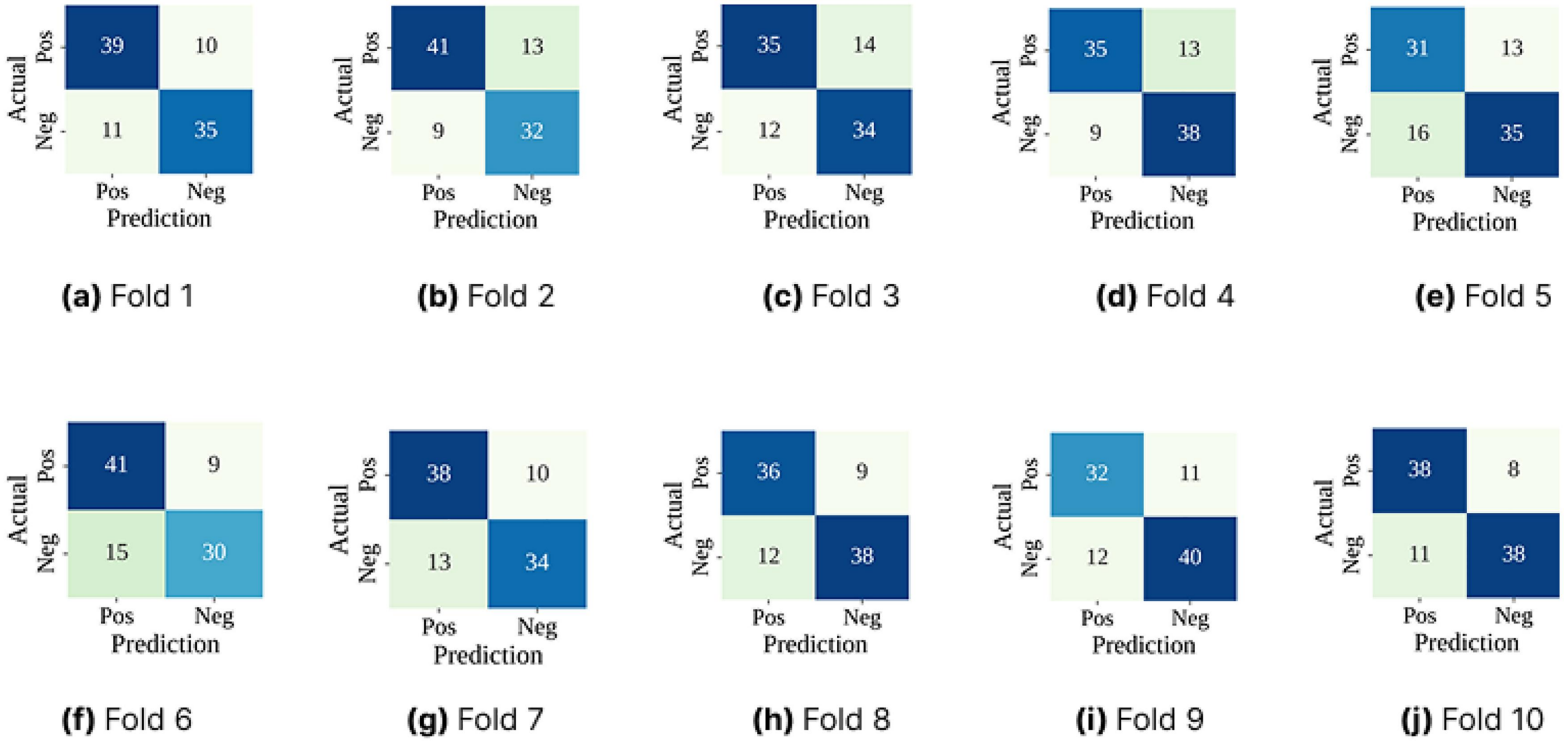
Confusion matrices (CM) across 10-folds using RoF-BESA with cross-validation, where **(a)** Fold 1 CM, **(b)** Fold 2 CM, **(c)** Fold 3 CM, **(d)** Fold 4 CM, **(e)** Fold 5 CM, **(f)** Fold 6 CM, **(g)** Fold 7 CM, **(h)** Fold 8 CM, **(i)** Fold 9 CM, and **(j)** Fold 10 CM.

The confusion matrices for each fold are summarized in Figure 6. Compared to our baseline model, RoF-BESA showed fewer misclassifications. Overall the classifier performed consistently well with only a small number of cases being misclassified in each fold. Importantly the number of false negatives i.e., gallstone cases predicted as healthy was kept low. This is critical in a medical setting. Although there were slight differences from fold to fold, the overall trend shows that the optimized model was stable and reliable in its predictions.

**Figure 7:**
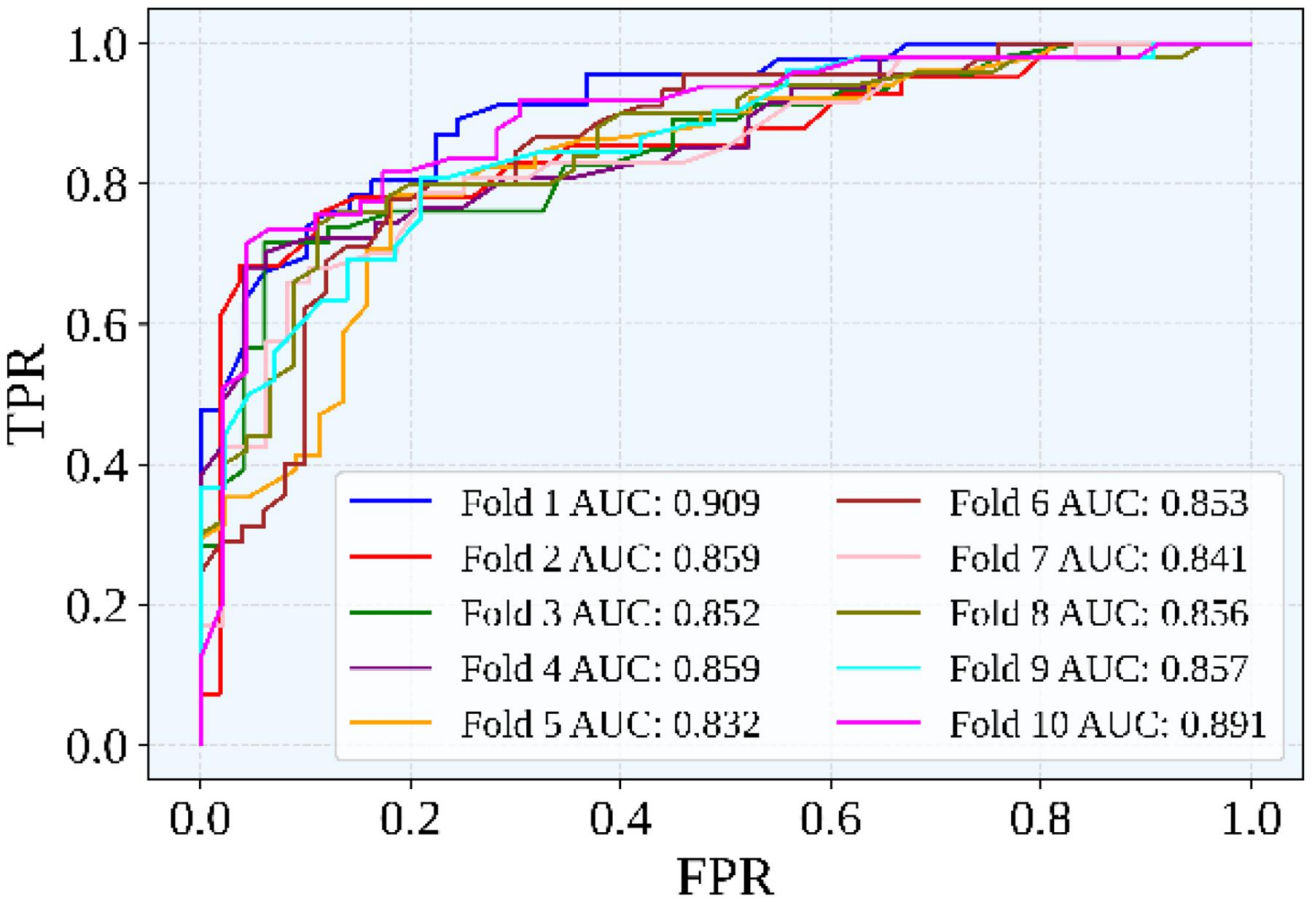
Combined ROC curve for all 10 folds.

From Figure 7, it is clear that the AUC values across folds range from 0.832 to 0.909. This indicates consistently strong classification performance and robustness of the model. The optimized classifier achieved an average AUC of 0.8609. This data demonstrates a better ability to distinguish between classes and more consistent performance than the baseline model. Overall, BES optimization strengthened the rotational forest classifier by ensuring more consistent performance across folds and reducing key misclassifications.

#### 3.2.3 SHAP and LIME analysis

SHAP analysis was performed to better understand the predictions of the optimized rotational Forest model. This method helps reveal how each feature contributes to the model's decisions, both for individual patients and across the dataset as a whole.

**Figure 8:**
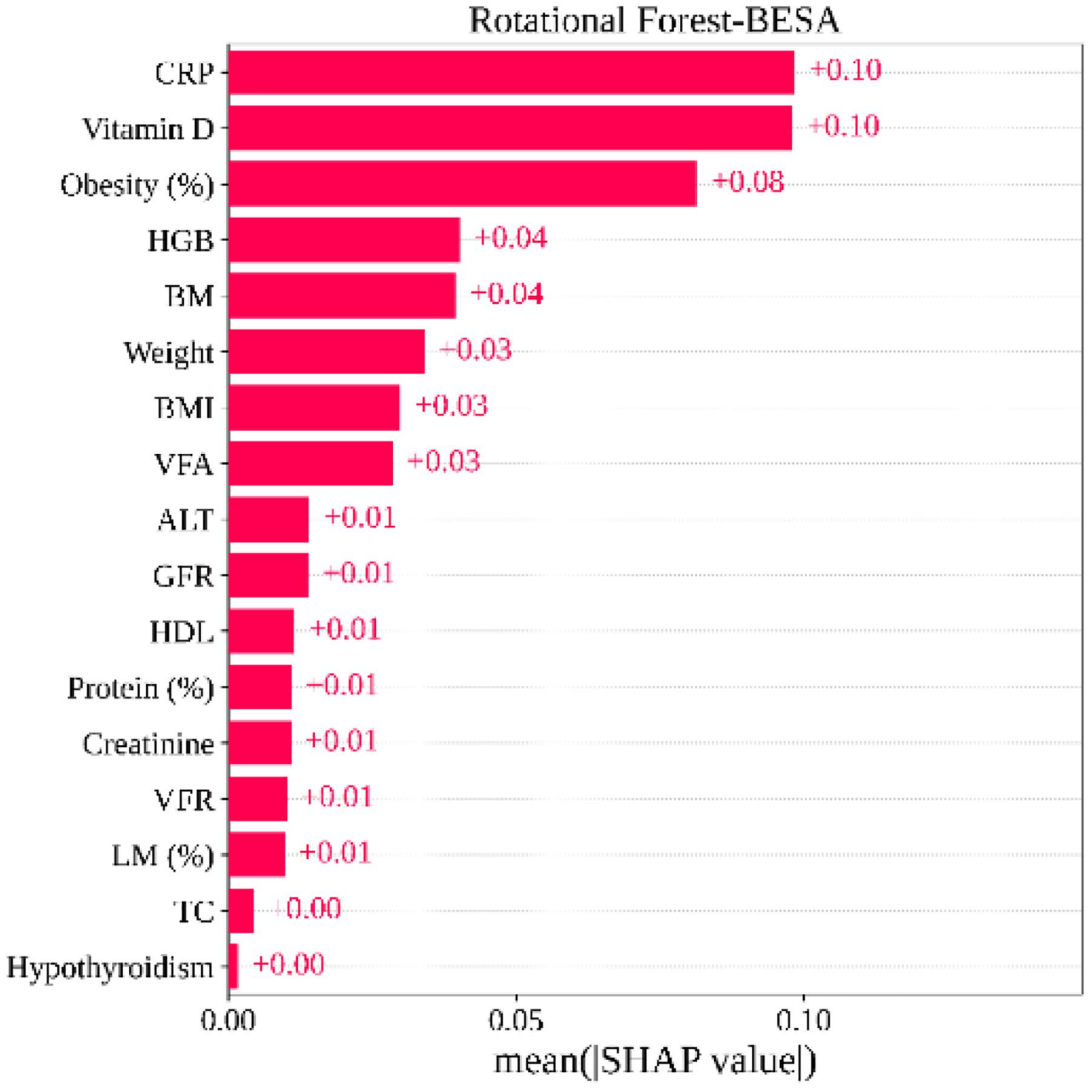
Important features along with their SHAP values.

**Figure 9:**
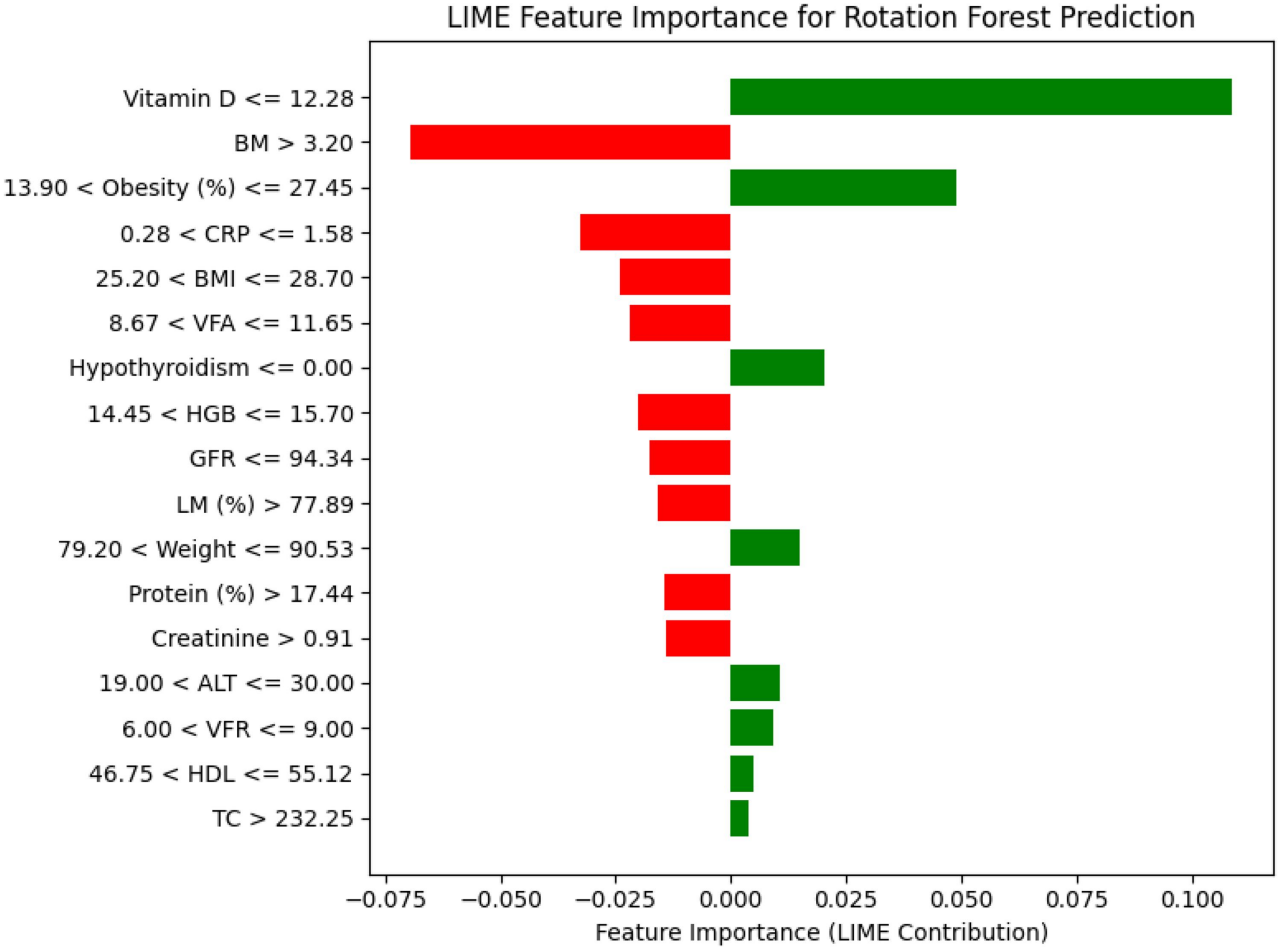
Important features using LIME.

Figure 8 shows the feature importance of the RoF-BESA model. Among the selected features, CRP has shown the highest impact on the model with a mean SHAP value of +0.10. Vitamin D and Obesity have been found in the 2nd and 3rd place in the feature ranking, with the mean SHAP value of +0.10 and +0.08. HGB (+0.04) and BM (+0.04) are the next two features of the top five influential features. These results present both the overall feature importance and patient-level insights. This provides a deeper understanding of the patterns for gallstone prediction. The LIME findings in Figure 9 indicate that Vitamin D, BM, Obesity, CRP, and BMI are the top five features influencing the model's predictions.

## 4 Discussion and future work

In this study, we have implemented two methods to predict individuals with a high risk of gallstones using a tabular dataset. In the first method, we applied the RoF classifier without any optimization to understand the model's natural performance. In the second method, we enhanced the classifier by optimizing it with BESA. This helped in selecting the most relevant features as well as the hyperparameters. This RoF-BESA model reduced critical misclassifications, making it more reliable. We also used SHAP analysis to even better understand how the models make predictions and to interpret the contribution of each feature. Our RoF-BESA model becomes not only more accurate and stable but also transparent due to the combination of optimization with interpretability.

The comparison in Table 4 shows how our models performed against previously published studies using random forest for gallbladder prediction. Prior works reported accuracies between about 78% and 96%, depending on whether they used tabular data or medical images. The image-based study (26) showed the highest accuracy (96%) and AUC (0.988). The reason for this is that images often provide richer information than tabular records. For tabular datasets, most random forest models reported accuracies in the range of 78%–85% with AUC values from 0.71 to 0.85. Notably, Esen et al. (11) obtained an accuracy of 85.42% but their model relied on a larger feature set of 38 variables. On comparison, our models achieved 75%–78% accuracy and an AUC of 0.860–0.867 with a feature set of 17 variables. In terms of computational efficiency, the mean testing times of our models were 0.00945 s (RoF alone) and 0.00946 s (RoF with BESA), respectively, indicating nearly identical performance. Both of our models attain the highest AUC for the tabular dataset. This demonstrates improved discrimination compared to other models using the tabular dataset. This suggests that our approach is both effective and competitive for gallstone prediction using tabular data. Similarly, most of the previous studies use random forest on private datasets (13, 14, 16). In contrast, we applied RoF, which introduces feature-space rotations to improve classifier diversity and performance.

**Table 4:** Performance comparison.

Work	Dataset type	Model used	Accuracy (%)	F1 score (%)	Precision (%)	Recall (%)	AUC
(27)	Tabular	Random Forest	-	78.95	78.40	79.50	0.853
(11)	Tabular	Random Forest	85.42	86	91	81	0.85
(28)	Tabular	Random Forest	78.2	-	-	62.6	0.71
(26)	Image	Random forest	96	96.36	95.42	97.32	0.988
Our models							
Our	Tabular	RoF	78	77.350	79.331	75.795	0.867
Our	Tabular	RoF-BESA	75.789	75.411	76.323	74.700	0.860

Based on the performance metrics, our RoF model is performing better than the RoF-BESA model. However, RoF-BESA has used fewer features compared to RoF. This reduction in features makes the model more efficient, less prone to overfitting, and easier to interpret. This is an important factor for the prediction of cholelithiasis. RoF-BESA not only simplifies computation but also highlights potential key risk factors associated with gallbladder conditions by narrowing down to the most relevant variables. Thus, even though RoF appears stronger in raw accuracy, RoF-BESA provides a more balanced solution. It offers reliable predictions while being faster and easier to understand, which is especially important in real healthcare use.

In summary, our RoF model matches the accuracy of existing work, and our RoF-BESA provides a strong balance between performance and simplicity. To the best of our knowledge, this is the first study to apply BESA with RoF for gallbladder prediction. This also shows its potential to support both accurate and interpretable medical decision-making.

The LIME shows that Vitamin D, BM, Obesity, CRP, and BMI are the top five features influencing the model's predictions. Similarly, the SHAP explanations identify CRP, Vitamin D, Obesity, HGB, and BM as the most impactful features. These results show that both XAI techniques highlight largely overlapping feature importance, confirming the consistency of the model's interpretability across different explanation methods. These supplementary findings demonstrate that the model's interpretability remains consistent across different XAI techniques, while ensuring that the main manuscript remains aligned with the intended design of the proposed framework.

Clinically, our model has meaningful implications for improving gallstone diagnosis. The SHAP-based analysis has identified the most influential features, such as (CRP, Vitamin D, Obesity), enabling clinicians to understand which clinical indicators play a critical role for individual patients. Additionally, reducing the feature set from 38 to 17 optimized features reduces unnecessary tests, supporting faster, more efficient decision-making. Overall, this model is a reliable and useful tool for improving gallstone classification.

Despite these promising results, there are also several limitations to consider. The major limitation lies in the relatively small (319 cases) dataset, and this might reduce the model's consistency and robustness. Our study is based on a single dataset, so its findings might not generalize to different groups of patients. Future work can address these limitations by including larger and more diverse datasets to improve generalizability. Adding other types of data, such as imaging or genetic information, could enhance prediction accuracy and clinical value. Exploring alternative optimization techniques may also further improve feature selection and model performance.

## 5 Conclusion

Gallstone disease is a common gastrointestinal disorder that affects millions worldwide and can lead to serious complications if left untreated. Early and accurate prediction of gallstones is therefore crucial to prevent unnecessary surgeries, reduce healthcare costs, and improve overall patient outcomes. In this study, we developed an interpretable and optimized rotational forest framework using the bald eagle search algorithm (RoF-BESA) for gallstone prediction. Our baseline rotational forest model achieved an accuracy of 78%, F1 score of 77.35%, precision of 79.33%, recall of 75.79% and AUC of 0.86. Our RoF-BESA model attained an accuracy of 75.789%, F1 score of 75.41%, precision of 76.323%, recall of 74.700%, and AUC of 0.860 with 17 feature sets. The RoF-BESA not only simplifies the model but also makes it faster and less prone to overfitting by reducing the feature size to 17 key variables. This is an essential factor in healthcare, where understanding why a prediction is made can be as important as the prediction itself. The model not only showed stable, reliable performance but also minimized misclassifications. It also identified the most important predictive features using SHAP analysis. This approach highlights the potential of combining metaheuristic optimization with explainable AI for practical clinical decision support.

## Data Availability

Publicly available datasets were analyzed in this study. The datasets analyzed for this study can be found in the UCI machine learning repository, https://archive.ics.uci.edu/dataset/1150/gallstone-1.
